# American Academy of Dental Science

**Published:** 1870-12

**Authors:** Edward N. Harris

**Affiliations:** Cor. and Rec. Sec.


### ARTICLE V.

### *American Academy of Dental Science.*

The Third Annual Meeting of the American Academy of Dental Science was held in Mercantile Hall, Boston, September 26th 1870, at 12 o'clock, M.

The President, Dr. Daniel Harwood, on taking the chair, congratulated the members upon the present prosperous condition of the Academy, and upon the encouraging prospects for the future.

There was a good attendance of members present, including a number from other States, among whom were Dr. J. H. Foster of New York, Dr. F. N. Seabury, of Providence, R. I., Dr. Elbridge Bacon, of Portland, Maine, Dr. C. K. Fiske of St. John, N. Brunswick, and Dr. Edward Gage, of Paris.

The following officers were elected for the ensuing year:

*President*, Daniel Harwood, M. D.

*Vice President*, E. T. Wilson, M. D.

*Cor'g and Rec'g Secretary*, E. N. Harris, D. D. S.

*Treasurer*, E. G. Tucker, M. D.

*Librarian*, John Clough, M. D.

*Board of Censors*, Drs. E. G. Tucker, J. L. Williams, and W. W. Codman.

At this and the previous monthly meeting, the following, new members were admitted:

Drs. T. B. Hitchcock, L. D. Shepard, and T. H. Chandler, Professors in the Harvard Dental College, Boston, were elected active fellows; also Drs. J. T. Codman, of Boston, J. H. Batchelder, of Salem, and S. P. Martin, of Worcester.

Dr. F. N. Seabury, T. D. Thompson, M. B. Mead, of Providence R. I., James McManus, of Hartford, Conn., and H. M. Bowker, of Montreal, Canada, were elected Associate Fellows.

Dr. Amos Westcott, of Syracuse N. Y. was elected an Honorary Fellow.

The Annual Address was delivered by Joseph H. Foster, M. D., of New York, on the subject of "Professional Responsibilities." Dr. Foster occupied one hour and twenty minutes in the delivery of his address, which was a very able production, and eloquently delivered, and received the hearty applause of the assembly.

The thanks of the Academy were presented to Dr. Foster, and a copy of his address requested for publication and for preservation in the Archives of the Society.

Interesting papers were read by Dr. Joseph E. Fisk of Salem, on "The Manufacture of Mineral Teeth"—By H. F. Bishop, D. D. S., of Worcester, on "Dental Societies"—and By John Clough, M. D., of Woburn, on "Causes of Diseased Teeth and Gums, and Preventive Measures for their Preservation."

At half past 5 P. M. the Academy adjourned to the Parker House to partake of the third Anniversary Dinner, which was a very elegant affair, served in Parker's usual excellent style, and the festivities were continued until a late hour.

EDWARD N. HARRIS, D. D. S.

Cor. and Rec. Sec.

---

### SELECTED ARTICLES.

---

